# Supplementary material for: Chromothripsis during telomere crisis is independent of NHEJ, and consistent with a replicative origin
Source: Genome Res. 2019 May;29(5):737–49. doi: 10.1101/gr.240705.118 (PMC6499312; doi:10.1101/gr.240705.118)
Supplement: Supplemental Material [file supp_gr.240705.118_Supplemental_file_1.zip › contigs/annotated_contigs/DB79/contig.2.DB79_length_293_mean_cov_5.63139931741.docx]

**DB79_length_293_mean_cov_5.63139931741**

```
GTAGTTGGAGGCTGTGCCAGCTTGGGGTGGGGCCACCTCAGTGTTCCTTTTTTTTGAGACAGAGTCTCACTCTGTCACCCAGGCTGGAG
 >chr11:61970009-61970108 - E=2e-43
TTCA|GTGGC|AGCACCATTTTACACCCCCATTCCTGCCAGTAATATATGAGGGTACAGAGAGCTCTTTTGGTGGCACCTAAGAACTGT
     >chr7:141809199-141809400 - E=5e-105
AAATAACAGTGTTCACAGTAGTGAATCTTTGTGTTTGCTTCAAATGCTCAGTATGTGGAAAAGCACACATACACACACACACGGAGAGA

GAGAGAGAAAGTGAGAGAGAGAGAGGGA
```
